# Nosological Differences in the Nature of Punctate White Matter Lesions in Preterm Infants

**DOI:** 10.3389/fneur.2021.657461

**Published:** 2021-04-29

**Authors:** Mariya Malova, Elena Morelli, Valentina Cardiello, Domenico Tortora, Mariasavina Severino, Maria Grazia Calevo, Alessandro Parodi, Laura Costanza De Angelis, Diego Minghetti, Andrea Rossi, Luca Antonio Ramenghi

**Affiliations:** ^1^Neonatal Intensive Care Unit, IRCCS Istituto Giannina Gaslini, Genoa, Italy; ^2^Neuroradiology Unit, IRCCS Istituto Giannina Gaslini, Genoa, Italy; ^3^Epidemiology and Biostatistics Unit, IRCCS Istituto Giannina Gaslini, Genoa, Italy

**Keywords:** punctate white matter lesions, brain damage, preterm, magnetic resonance imaging, newborn, SWI

## Abstract

**Background:** The pathogenesis of punctuate white matter lesions (PWMLs), a mild form of white matter damage observed in preterm infants, is still a matter of debate. Susceptibility-weighted imaging (SWI) allows to differentiate PWMLs based on the presence (SWI+) or absence (SWI–) of hemosiderin, but little is known about the significance of this distinction. This retrospective study aimed to compare neuroradiological and clinical characteristics of SWI+ and SWI– PWMLs.

**Materials and Methods:** MR images of all VLBW infants scanned consecutively at term-equivalent age between April 2012 and May 2018 were retrospectively reviewed, and infants with PWMLs defined as small areas of high T1 and/or low T2 signal in the periventricular white matter were selected and included in the study. Each lesion was analyzed separately and characterized by localization, organization pattern, and distance from the lateral ventricle. Clinical data were retrieved from the department database.

**Results:** A total of 517 PWMLs were registered in 81 patients, with 93 lesions (18%) visible on SWI (SWI+), revealing the presence of hemosiderin deposits. On univariate analysis, compared to SWI- PWML, SWI+ lesions were closer to the ventricle wall, more frequently organized in linear pattern and associated with lower birth weight, lower gestational age, lower admission temperature, need for intubation, bronchopulmonary dysplasia, retinopathy of prematurity, and presence of GMH-IVH. On multivariate analysis, closer distance to the ventricle wall on axial scan and lower birth weight were associated with visibility of PMWLs on SWI (*p* = 0.003 and *p* = 0.0001, respectively).

**Conclusions:** Our results suggest a nosological difference between SWI+ and SWI– PWMLs. Other prospective studies are warranted to corroborate these observations.

## Introduction

Following a noteworthy reduction in the incidence of severe forms of white matter injury in preterm infants, modern research now focuses on mild forms of damage, such as punctate white matter lesions (PWMLs). PWMLs are diagnosed in up to 30% of very preterm infants (1–6) and can be defined upon brain magnetic resonance imaging (MRI) as focal areas of high T1 and/or low T2 signal intensity located in the periventricular white matter (7).

The pathophysiology of PWMLs is still a matter of debate (6, 7). They are often considered ischemic/inflammatory in origin (8, 9); however, since their first description, it has been postulated that at least some punctate lesions can be hemorrhagic (8). These PWMLs are reported to be connected with congested medullary veins, and their pathogenesis could be similar to that of small venous infarcts (9, 10). Susceptibility-weighted imaging (SWI), a recently developed MRI sequence highly sensitive to hemosiderin deposits, can detect hemorrhagic PWMLs (visible as punctate areas of decreased signal, SWI+), distinguishing them from lesions without hemosiderin (not visible, SWI–) (9, 10). In a previous study, we have found evidence regarding the diversified clinical risk factors of SWI+ and SWI– PWMLs, suggesting that these two lesions have different pathogenesis (6).

To corroborate the hypothesis of the different nature of SWI+ and SWI– lesions, the aim of the present study was to further characterize these two types of PWMLs based on their anatomic localization, pattern, and associated clinical factors.

## Materials and Methods

In April 2012, routine brain MRI at term-equivalent age (TEA, between 39 + 0 and 41 + 6 weeks post-menstrual age) was introduced as part of a screening program for prematurity-related lesions in all very low birthweight (VLBW) infants admitted to our department. For the present study, MR images of all VLBW infants scanned consecutively between April 2012 and May 2018 were retrospectively retrieved and reviewed by two neuroradiologists experienced in neonatal neuroimaging (performing more than 200 neonatal brain MRI per year: MS and DT). Infants presenting PWMLs, defined as small areas of high T1 and/or low T2 signal in the periventricular white matter (7), were selected and included in the study. The presence of germinal matrix–intraventricular hemorrhage (GMH–IVH), cerebellar hemorrhage (CBH) and cystic periventricular leukomalacia (PVL) on MRI scans of included subjects was registered. The clinical data of the enrolled patients, including antenatal steroid course, type of delivery, birth weight, gestational age, need for intubation, presence of bronchopulmonary dysplasia (BPD), retinopathy of prematurity (ROP) and of necrotizing enterocolitis (NEC) were retrieved from clinical charts. This retrospective study was approved by the local ethics committee.

### Image Acquisition

MRI was performed at TEA during spontaneous sleep using the “feed and wrap” technique. The need for sedation (oral midazolam, 0.1 mcg/kg) to prevent head motion was determined by the neuroradiologist based on the quality of the images after the first sequence and the infants' state of arousal. Hearing protection was used in all patients, and heart rate and oxygen saturation were monitored non-invasively. Scans were performed using a 1.5 Tesla MR system (InteraAchieva 2.6; Philips, Best, The Netherlands) with a dedicated pediatric head/spine coil. Institutional standard MRI protocol in use at the moment of the study included 3-mm thick axial T2- and T1-weighted images, coronal T2-weighted images, sagittal T1-weighted images, axial diffusion-weighted images (b value: 1,000 s/mm^2^), and axial SWI. Informed consent, including statements regarding the significance and limitations of MRI at TEA, was obtained in all cases.

### Image Analysis

**Figure 1:**
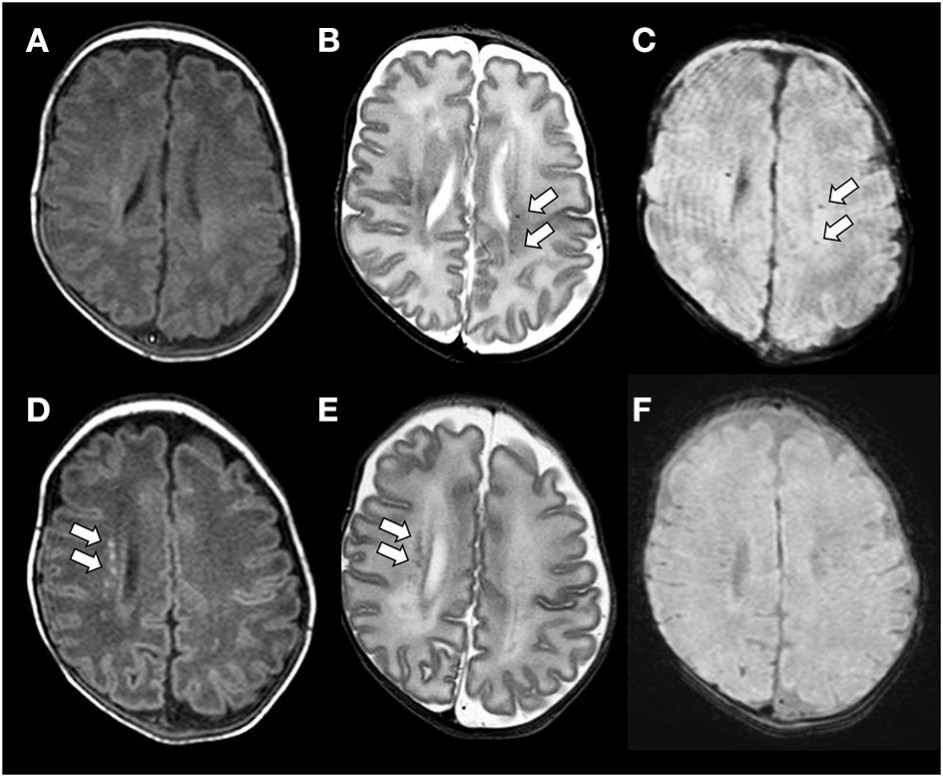
**(A–C)** Brain magnetic resonance imaging (MRI) performed at the term-equivalent age of an ex-preterm infant born at 25 weeks of gestation showing SWI+ PWMLs. SWI+ lesions are barely detectable on the axial T1-weighed image **(A)**, whereas they are clearly visible as hypointense spots on axial T2-weighted **(B)** and SWI **(C)** sequences. **(D–F)** Brain MRI performed at term-equivalent age of an ex-preterm infant born at 29 weeks of gestation showing SWI– PWMLs. SWI- lesions are more clearly defined on the axial T1-weighted **(D)** than on the axial T2-weighted image **(E)** and are not visible on SWI **(D)**.

A secondary analysis of MR scans of included subjects was performed by two trained investigators (EM and MM) blinded to the clinical history of the patients. Each PMWL was analyzed separately. In the presence of a low signal on SWI (“black dot”), corresponding to hemosiderin deposits, PWML was defined as SWI+, whereas in the absence of a low signal, it was defined as SWI– (10) (Figure 1).

The distance between each lesion and the ventricle was measured on axial (T1 or T2, based on major visibility) and coronal (T2) scans using the “measure” instrument of the PACS visualizer (Carestream Vue Motion, Carestream Health, Inc. 2018). The distance was measured using a straight line perpendicular to the ventricle wall.

Anatomic position on the axial scan of each lesion was determined as defined in the literature (8):

- anterior region (anterior to the frontal horn of the lateral ventricles)
- mid-region (in between or in the centrum semiovale)
- posterior region (posterior to the occipital horn of the lateral ventricles).

Each lesion was then characterized based on the pattern formed with the surrounding lesions [adapted from (7, 8)]:

- cluster (organized in a confluent pattern)
- linear (organized linearly)
- singular (not in proximity with other lesions).

### Statistical Analysis

Descriptive statistics were generated for the whole cohort and data were expressed as mean and standard deviation for continuous variables. Median value and range were calculated and reported, as were absolute or relative frequencies for categorical variables. Clinical characteristics were compared using the χ^2^ or Fisher's exact test and the Student *t*-test for categorical and continuous variables, respectively. Logistic regression analyses were used, and the results were reported as odds ratio (OR) with their 95% confidence intervals (CIs). The absence of exposure to the factor or the variable that was less likely to be associated with the risk of the lesion was used as the reference for each analysis. Multivariate analysis was then performed, and only variables that proved to be statistically or borderline significant in univariate analysis (*P* < 0.08) were included in the model. The model showing the best fit was based on backward stepwise selection procedures, and each variable was removed if it did not contribute significantly. In the final model a *P*-value of < 0.05 was considered statistically significant, and all *P*-values were based on two-tailed tests. Statistical analysis was performed using Statistical Package for the Social Sciences (SPSS) for Windows (SPSS Inc., Chicago, IL).

## Results

During the study period, 428 VLBW infants admitted to the Neonatal Intensive Care Unit of IRCSS Istituto Giannina Gaslini underwent brain MRI at TEA. Eighty-one (19%) of them presented with PWMLs and were included in the study. The gestational age at birth of the included subjects ranged between 24 and 34 weeks (mean 28.6 ± 2.1 weeks) and the birth weight ranged between 470 and 1,495 g (mean 1,140 ± 240 g). In 50 infants out of 81 PWMLs were an isolated finding, while remaining 31 patients presented one or more additional prematurity-related brain lesions (GMH-IVH in 26 cases, CBH in 13 and cystic PVL in 6).

A total of 517 PWMLs were registered in 81 patients. Ninety-three lesions (18%) presented a low signal on the SWI sequence, consistent with the presence of hemosiderin deposits, and were therefore classified as SWI+. The remaining 424 lesions (82%) were classified as SWI–.

Clinical parameters distribution in the groups of SWI+ and SWI– lesions are summarized in Table 1. SWI+ PWMLs were characterized by lower mean gestational age and birth weight, lower admission temperature, higher frequency of need for intubation, of GMH–IVH, ROP and BPD (all *p* < 0.001).

**Table 1:** Clinical parameters in the groups of SWI+ and SWI– PWMLs.

	**SWI+ PWMLs (*N* = 93)**	**SWI– PWMLs (*N* = 424)**	***p*-value**
Birth weight (gr), mean ± SD	1,000 ± 282	1,202 ± 186	**<0.001**
median (range)	870 (470; 1,495)	1,230 (780; 1,495)	
Gestational age (weeks), mean ± SD	27.4 ± 2.53	29 ± 1.76	**<0.001**
median (range)	28 (24; 34)	29 (25; 34)	
Male gender	43 (46.2%)	221 (52.1%)	0.36
Absent or incomplete antenatal steroid treatment	26 (28%)	164 (38.7%)	0.06
Histological chorioamnionitis^*^	6 (12%)	68 (23.7%)	0.07
Vaginal birth	31 (33.3%)	152 (35.8%)	0.72
Apgar score at 5° min: mean ± SD	7.8 ± 1.3	8.1 ± 0.9	0.34
Admission temperature: mean ± SD	35.8 ± 0.38	36.2 ± 0.46	**<0.001**
Need for intubation	86 (92.5%)	316 (74.5%)	**<0.001**
GMH-IVH	85 (91.4%)	104 (24.5%)	**<0.001**
Retinopathy of prematurity	64 (68.8%)	159 (37.5%)	**<0.001**
Necrotizing enterocolitis	12 (12.9%)	47 (11.1%)	0.59
Bronchopulmonary dysplasia	41 (44.1%)	33 (7.8%)	**<0.001**

* *Data on chorioamnionitis available for 337/517 lesions. PWMLs, punctate white matter lesions; SWI, susceptibility-weighted imaging; GMH–IVH, germinal matrix hemorrhage–intraventricular hemorrhage*. *Bold was used to evidence p < 0.05, corresponding to the statistically significant results*.

When anatomic distribution was considered, SWI+ lesions were located significantly closer to the ventricle wall than SWI– lesions both on coronal and axial scans (Table 2). On the axial plane, both SWI+ and SWI– lesions were predominantly located in the mid-region as compared with the anterior or posterior region, without significant differences among two types of PWMLs (Table 2). Most of the PWMLs were organized in linear pattern, with a significantly higher percentage of SWI+ lesions presenting with this pattern as compared with SWI– lesions (75.3 vs. 60.8%, *p* = 0.009) (Table 2).

**Table 2:** Neuroradiological characteristics of SWI+ and SWI– PWMLs.

	**SWI+ PWMLs (*N* = 93)**	**SWI– PWMLs (*N* = 424)**	***p*-value**
**Distance from the ventricle wall**			
Coronal scan (mm), mean ± SD	3.15 ± 1.93	4.79 ± 1.86	**<0.001**
Axial scan (mm), mean ± SD	3.75 ± 1.53	5.22 ± 2.17	**<0.001**
**Distribution on the axial plane**			
Anterior region	3 (3.2%)	7 (1.7%)	0.39
Mid-region	77 (82.8%)	376 (88.7%)	0.12
Posterior region	13 (14%)	41 (9.7%)	0.26
**Patterns of organization**			
Linear pattern	70 (75.3%)	258 (60.8%)	**0.009**
Cluster pattern	11 (11.8%)	84 (19.8%)	0.08
Singular lesion	12 (12.9%)	82 (19.3%)	0.18

*PWMLs, punctate white matter lesions; SWI, susceptibility-weighted imaging*.

*Bold was used to evidence p < 0.05, corresponding to the statistically significant results*.

Multivariate analysis including neuroradiological and clinical parameters showed that the distance from the ventricle on axial plane and birth weight were associated with the type of PWMLs (Table 3).

**Table 3:** Results of multivariate analysis including clinical and neuroradiological characteristics of SWI+ and SWI– PWMLs.

	**SWI+ PWMLs (*N* = 93)**	**SWI– PWMLs (*N* = 424)**	**OR (95%CI)**	***p*-value**
Distance from the ventricle on axial scan (mm), mean ± SD	3.75 ± 1.53	5.22 ± 2.17	0.73 (0.59–0.89)	**0.003**
Birth weight (gr), mean ± SD	1,000 ± 282	1,202 ± 186	0.994 (0.992–0.996)	**0.0001**

*OR, odds ratio; CI, confidence interval; PWMLs, punctate white matter lesions; SWI, susceptibility-weighted imaging*.

*Bold was used to evidence p < 0.05, corresponding to the statistically significant results*.

## Discussion

In this retrospective study we analyzed anatomic distribution, pattern, and correlation with the clinical data of PWMLs positive and negative on SWI. In our study, 18% of PWMLs presented with decreased signals on the SWI sequence, corresponding to the presence of hemosiderin deposits within the lesion compatible with petechial/hemorrhagic pathogenesis (8, 10). The precise pathologic mechanism behind the development of SWI+ PWMLs remains unknown, but we could speculate that venous sludging and microthrombosis in the medullary veins of the periventricular white matter could play a role, as has been suggested for the pathogenesis of GMH–IVH (11–13). Accordingly, GMH–IVH is more common in babies with inherited thrombophilia; however, we did not investigate any gain-of-function gene mutations like Prothrombin G20210 or Arg506Gln Factor V Leiden, as compared with our previous studies (14). Deep medullary vein congestion has been associated with different patterns of white matter damage in a single-center MRI imaging study (15) as well as in previous observations of infants with GMH–IVH due to cerebral sinovenous thrombosis (16, 17). The hemosiderin deposits within SWI+ PWMLs may act as proinflammatory stimuli for the adjacent white matter as well as interfere with myelination (18, 19).

In this work we observed that SWI+ PWMLs are significantly closer to the ventricular wall, further confirming their possible connection with the congested veins (9). This observation could be explained by the anatomic distribution of the medullary veins flowing vertically into the subependymal veins near the ventricle as well as by their relative immaturity and fragility in a premature infant (20). Interestingly, the distance from the ventricle on axial scan was associated with PWML type independently of clinical parameters.

On the other hand, SWI– PWMLs are distributed further away from the ventricular wall in zones prone to inflammation due to active myelination that are often targeted by cystic PVL (21). A historical work by Leech and Alvord considering the morphologic variations in PVL included the description of focal white matter lesions across the entire depth of the white matter (“Type 1”), with less intense lesions further away from the ventricle (22). Although there is a lack of widespread histological studies of PWML, punctate lesions and PVL are often seen as extremes of the same spectrum (7, 9). Accordingly, the MR T1 signal around developing PVL cavitation is similar in intensity to the signal of SWI– PWMLs. We thus hypothesize that SWI– PWMLs represent the milder form of PVL, sharing its ischemic/inflammatory pathogenesis (9).

Concerning the distribution of PWMLs on the axial scan, in our population, the majority of PWMLs (82.8% of SWI+ and 88.7% of SWI– lesions) were located medially along the lateral ventricles, as reported in the literature (7–9). We did not observe a significant difference in the anatomic distribution between SWI+ and SWI– lesions on the axial scan, although SWI+ lesions were slightly more frequently located frontally and posteriorly compared with SWI– lesions. It remains uncertain whether a different lesion pathophysiology could have contributed to this slight difference, with hemorrhagic lesions being more ubiquitous and not connected with specific inflammation-prone areas of the white matter, such as the corona radiata.

We investigated the distribution patterns of punctate lesions (linear, cluster, or singular), as adapted from previous studies (7, 8). In our study, compared with SWI– lesions, SWI+ lesions were more frequently distributed in the linear pattern (60.8 vs. 75.3%). Similar results were observed by Kersbergen et al., who reported a significant association between the linear appearance of lesions and low signal intensity on SWI. The proximity of SWI+ lesions to the medullary veins, distributed in a comb-shape appearance on axial view could have played a role in this association.

Different clinical factors were investigated in relation to PWMLs; however, only few were found to be significant. These include higher gestational age, greater birth weight, and the presence of IVH grade II or III (4, 23). In our population, GMH-IVH was the most frequent additional prematurity-brain lesion observed, even if in major part of infants PWMLs were an isolated finding. In term infants, congenital heart diseases and surgery for non-cardiac congenital anomalies have been linked to PWMLs (24, 25). We have recently shown that birth weight, absent or incomplete antenatal steroid course, vaginal delivery, and the need for intubation are risk factors of the different types of PWMLs in preterm infants (6). In the present work, we analyzed the distribution of these and some other clinical factors in the groups of PWMLs visible and not visible on SWI. We observed a significantly lower mean gestational age at birth and a smaller mean birth weight in the SWI+ PWML group than in the SWI– PWML group, with birth weight remaining significant on multivariate analysis. These differences could reflect the higher vulnerability of the extremely premature brain to hemorrhagic lesions (26), including SWI+ PWMLs. Progressive maturation of the medullary veins with growing gestational age could play a protective role as well (18). At the microscopic level, the astrocyte-dependent structural integrity of white matter vessels depends, at least in part, on gestational age (27). It would be interesting to know whether the immature blood–brain barrier in infants with lower gestational ages can favor blood transudation of blood by-products with the subsequent formation of SWI+ PWMLs. However, widespread histological studies on this topic are lacking, partially due to the absence of an animal model.

Accordingly, SWI– PWMLs are associated with higher gestational age and higher birth weight, similar to PVL (9). Specific white matter vulnerability to damage in this phase of maturation could be related to the presence of maturating oligodendrocyte precursors (28) as well as to major microglial activation, with subsequent alterations in myelination and white matter injury (29).

Other clinical factors observed more frequently in SWI+ group, as higher need for intubation, lower admission temperature and more frequent presence of ROP, BPD and GMH-IVH, are probably secondary to lower gestational age and birth weight, even if an association between SWI+ PWML and GMH-IVH has been previously described in the literature (10).

The prognostic significance of PWMLs remains debatable. Some cases seem to be followed by motor disability, cerebral palsy, or altered cognitive performance, while others are accompanied by normal neurodevelopment (2, 4, 5, 7, 30). In a study involving 12 infants with isolated PWMLs followed up until school age, a higher risk of dyspraxia and motor impairment was observed, without increased risk of cognitive impairment (31). In other studies, worse outcome after PWMLs was associated with higher lesion number (2, 4), cluster pattern organization (7), and frontal lobe distribution on axial imaging (5). It would be interesting to investigate the impact on neurodevelopment of PWMLs based on their visibility on SWI, and the related study is ongoing. Our work has several limitations. First, owing to the retrospective design of the study, the findings of our study need to be validated via prospective studies. Furthermore, we studied a very specific population (i.e., VLBW infants); therefore, the extension of our results to other patient groups can be questionable. This is important considering that PWMLs have been described at all gestational ages up to term age (9). In addition, the use of TEA MRI may have limited our ability to identify those PWML disappearing at an earlier stage (7), even if their clinical significance remains to be defined. Finally, the absence of early MRI did not allow us to use diffusion restriction as a marker of PWMLs with possible inflammatory or ischemic origin, as described in the literature (7).

On the other hand, the anatomic dispositions of punctate lesions were analyzed one by one, thereby allowing a more precise characterization of their different anatomic distributions. This represents a potential strength of our study. In addition, the availability of the SWI sequence in all MRIs since 2012 has allowed us to clearly identify PWMLs with the presence of hemosiderin (SWI+) as well as to analyze them separately from SWI– PWMLs.

In conclusion, in this study we observed statistically significant differences between PWMLs visible and not visible on SWI sequence, with closer distance from the ventricle on axial scan and lower birth weight being associated with SWI+ lesions. These observations, together with previously evidenced differences in risk factors, suggest that SWI+ and SWI– PWMLs could represent two distinct nosological entities with potentially different nature. Further prospective studies are warranted to corroborate our findings as well as to define the clinical significance of the two types of PWMLs.

## Data Availability Statement

The original contributions presented in the study are included in the article/supplementary material, further inquiries can be directed to the corresponding author/s.

## Ethics Statement

The study was approved by the local ethics committee (Comitato Etico Regione Liguria, Genoa, Italy). Written informed consent to participate in the study was provided by the participant's legal guardian/next of kin.

## Author Contributions

MM and LR contributed to study planning and design and manuscript writing. MM and EM contributed to the data and imaging analysis and to manuscript writing. VC, LD, DM, and AP contributed to patient recruitment, data collection and analysis, and manuscript writing. MS and DT collected and analyzed MRI data and contributed to manuscript writing. MC performed statistical analysis. AR contributed to interpretation of collected data and to manuscript writing. All authors participated in revising the manuscript and approved the final version before publication.

## Conflict of Interest

The authors declare that the research was conducted in the absence of any commercial or financial relationships that could be construed as a potential conflict of interest.
